# Spectrum of germline and somatic mitochondrial DNA variants in Tuberous Sclerosis Complex

**DOI:** 10.3389/fgene.2022.917993

**Published:** 2023-01-30

**Authors:** Krinio Giannikou, Katie R. Martin, Ahmad G. Abdel-Azim, Kaila J. Pamir, Thomas R. Hougard, Shefali Bagwe, Yan Tang, Jeffrey P. MacKeigan, David J. Kwiatkowski, Elizabeth P. Henske, Hilaire C. Lam

**Affiliations:** ^1^ Cancer Genetics Laboratory, Division of Pulmonary and Critical Care Medicine, Brigham and Women’s Hospital and Harvard Medical School, Boston, MA, United States; ^2^ Division of Hematology/Oncology, Cancer and Blood Disease Institute, Children’s Hospital Los Angeles, Los Angeles, CA, United States; ^3^ Pediatrics and Human Development, College of Human Medicine, Michigan State University, Grand Rapids, MI, United States; ^4^ Center for LAM Research and Clinical Care, Division of Pulmonary and Critical Care Medicine, Brigham and Women’s Hospital and Harvard Medical School, Boston, MA, United States

**Keywords:** mTORC1, buccal swab, angiomyolipoma, lymphangioleiomyomatosis, renal cell carcinoma

## Abstract

Tuberous Sclerosis Complex (TSC) is caused by loss of function variants in either *TSC1 or TSC2* and is characterized by broad phenotypic heterogeneity*.* Currently, there is limited knowledge regarding the role of the mitochondrial genome (mtDNA) in TSC pathogenesis. In this study, we aimed to determine the prevalence and spectrum of germline and somatic mtDNA variants in TSC and identify potential disease modifiers. Analysis of mtDNA amplicon massively parallel sequencing (aMPS) data, off-target mtDNA from whole-exome sequencing (WES), and/or qPCR, revealed mtDNA alterations in 270 diverse tissues (139 TSC-associated tumors and 131 normal tissue samples) from 199 patients and six healthy individuals. Correlation of clinical features to mtDNA variants and haplogroup analysis was done in 102 buccal swabs (age: 20–71 years). No correlation was found between clinical features and either mtDNA variants or haplogroups. No pathogenic variants were identified in the buccal swab samples. Using *in silico* analysis, we identified three predicted pathogenic variants in tumor samples: *MT-ND4* (m.11742G>A, p. Cys328Tyr, VAF: 43%, kidney angiomyolipoma), *MT-CYB* (m.14775T>C, p. Leu10Pro, VAF: 43%, LAM abdominal tumor) and *MT-CYB* (m.15555C>T, p. Pro270Leu, VAF: 7%, renal cell carcinoma). Large deletions of the mitochondrial genome were not detected. Analysis of tumors from 23 patients with corresponding normal tissue did not reveal any recurrent tumor-associated somatic variants. The mtDNA/gDNA ratio between tumors and corresponding normal tissue was also unchanged. Overall, our findings demonstrate that the mitochondrial genome is highly stable across tissues and within TSC-associated tumors.

## 1 Introduction

Tuberous Sclerosis Complex (TSC; MIM# 191100, 191092) is an autosomal dominant neurocutaneous tumor suppressor gene syndrome caused by loss of function pathogenic alterations in either *TSC1* or *TSC2* with a prevalence of 1:6–10,000 live births (Henske et al., 2016). Affected individuals develop tumors in multiple organs including the brain, kidney, lung, heart, liver, and skin. Most of these tumors are benign, with the exception of TSC-associated renal cell carcinoma (TSC-RCC), which are aggressive affecting ∼3% of patients (Henske et al., 2021). Patients also have TSC-associated neuropsychiatric disorders (TANDs), seizures, intellectual disability, and autism (Henske et al., 2016; Yu et al., 2018). Tumors in TSC arise due to biallelic inactivating alterations in either *TSC1* or *TSC2*, leading to hyperactivation of mTORC1 and extensive metabolic reprogramming to support aberrant cell growth and proliferation (Duvel et al., 2010; Peron et al., 2018). Germline alterations in *TSC2* or *TSC1* gene are often associated with a highly variable phenotype, even among affected members of a single-family. This is thought to be due to the chance nature of the number and distribution of second-hit events that lead to tumor formation (Martin et al., 2017; Peron et al., 2018). Patients with *TSC2* pathogenic variants tend to have a more severe phenotype compared to patients with *TSC1* genetic alterations (Jones et al., 1997). Mosaicism is common in TSC, affecting 10%–15% of patients with confirmed clinical diagnosis, and is also associated with a milder phenotype (Verhoef et al., 1999; Tyburczy et al., 2015a; Bessis et al., 2018; Giannikou et al., 2019; Manzanilla-Romero et al., 2021). Previous exome analyses of various TSC related tumors have revealed a stable genome with an extremely low somatic mutation burden and copy number alterations, consistent with the benign nature of these tumors (Tyburczy et al., 2015b; Giannikou et al., 2016; Martin et al., 2017).

The mitochondrial genome accumulates alterations ∼10 faster than the nuclear genome in an age-dependent manner, which may be related to the mitotic index and mutagenic exposure of a tissue (Taylor and Turnbull, 2005; Tuppen et al., 2010). Heteroplasmy and the highly polymorphic nature of the mitochondrial genome make the identification of disease-causing or disease-modifying variants challenging in the clinical setting (Wang et al., 2012). Previous studies have shown that germline mitochondrial alterations may modify an individual’s risk for cancer development (de Matos et al., 2019; Tasdogan et al., 2020). Other studies have investigated the association of mtDNA variants defining particular ethnic groups with cancer initiation and progression (Calvo et al., 2006; Tasdogan et al., 2020; Crooks et al., 2021; Orsucci et al., 2021). Accumulation of somatic mtDNA variants and alterations in mtDNA/gDNA content has also been associated with various cancer types, including breast, colorectal, ovarian, prostate, lung, kidney, and brain (Triska et al., 2019; Yuan et al., 2020; Gorelick et al., 2021). While some recent studies have highlighted that tumors are characterized by accumulation of mtDNA alterations (Triska et al., 2019; Crooks et al., 2021; Gorelick et al., 2021), other large studies suggest that the deleterious mtDNA variants that adversely impact mitochondrial function are observed at a lower frequency than expected due to negative selection during tumorigenesis (Ju et al., 2014; Stewart et al., 2015).

Prior studies have shown that the nuclear genome is highly stable in TSC (Tyburczy et al., 2015b; Giannikou et al., 2016; Martin et al., 2017). Though TSC is not a mitochondrial disease, mTORC1 increases mitochondrial ROS in proximity to the mitochondrial genome, which has limited DNA repair mechanisms suggesting that altered mitochondria metabolism contributes to TSC phenotype (Zinovkina, 2018; Fontana and Gahlon, 2020). mTORC1 activation results in reduced autophagy, disruption of mitochondrial dynamics, and contributes to the production of the oncometabolite fumarate in preclinical TSC models (Ebrahimi-Fakhari et al., 2016; Drusian et al., 2018; Crooks et al., 2021). Also, the majority of TSC-RCCs are characterized by low levels of succinate dehydrogenase B (SDHB) staining, an enzyme that plays a critical role in mitochondria suggesting disruption of Complex II and a possible decrease in mitochondrial content compared to the surrounding renal tissue (Yang et al., 2014; Henske et al., 2021).

There are no prior genetic studies investigating the impact of mitochondrial genomic alterations on TSC pathogenesis and disease risk. Herein, we hypothesized that alterations in the mtDNA might play a role in TSC pathogenesis and act as a novel disease modifier contributing to tumorigenesis. We applied high throughput and robust massively parallel sequencing (MPS) methods to identify germline and somatic mtDNA variants in a large set of 270 diverse tissue samples from 199 subjects with TSC and six healthy controls. Across all tissue types, we identified only three SNVs predicted by *in silico* algorithms to be pathogenic in tumor samples with VAF<50%. Small deletions and insertions (indels) were identified in regions known to have high sequence heterogeneity, while large deletions were not detected. Finally, mtDNA/gDNA ratios were unchanged in tumors compared to corresponding normal tissue types. In sum, our data highlight the striking stability of the mitochondrial genome in TSC.

## 2 Materials and methods

### 2.1 Sample collection

DNA samples isolated from buccal swabs were provided by the TSC Alliance. All subjects had a definite clinical diagnosis of TSC meeting the standard inclusion criteria (Northrup et al., 2021). Clinical information, provided by the TSC Natural History Database, was available for 92% of the subjects with buccal swab samples available for analysis (Table 1, Supplementary Table S1). No details were provided regarding the severity of the 22 primary clinical manifestations, which were recorded for most TSC patients in the TSC Natural History Database. Most cases were sporadic, whereas six individuals with TSC had a family history of TSC (two trios and one sibling duo; Supplementary Figure S2). Limited clinical data were available for the tumor samples, which were previously published (Giannikou et al., 2016; Martin et al., 2017; Giannikou et al., 2021).

**TABLE 1:** Summary of clinical data associated with buccal swab samples.

Clinical features	Patient cohort n = 102 (%)
**Age median (range), years**	31, (20–71)
Female	54/102 (53)
Male	44/102 (43)
Unknown	4/102 (4)
**Tuberous Sclerosis Complex**	
*TSC1* mutant	18/102 (18)
*TSC2* mutant	35/102 (34)
Unknown	49/102 (48)
**Skin manifestations**	
Facial angiofibromas	82/95 (86)
Hypomelanotic macules	71/91 (78)
Shagreen patches	48/91 (53)
Ungual fibromas	39/88 (44)
**Cardiac rhabdomyomas**	32/81 (40)
**Retinal harmatomas**	18/77 (23)
**Brain manifestations**	
Cortical tubers	87/92 (95)
Subependymal nodules (SEN)	61/66 (92)
SEGA	28/89 (31)
Epilepsy	83/95 (87)
Autism	18/74 (24)
Intellectual disability	23/38 (61)
**Kidney angiomyolipoma**	**66/92 (72)**
**Renal cysts**	58/88 (66)
**LAM disease**	18/47 (38)
**TAND**	
ADHD/Depression/psychiatric disorders	42/85 (49)

This study was conducted in compliance with Institutional Review Board performed by Ethical & Independent Review Services in accordance with the ethical standards of the responsible committee on human experimentation (institutional and national) and with the Helsinki Declaration of 1975, as revised in 2013.

### 2.2 Deep coverage amplicon MPS analysis

High-quality DNA was isolated from 102 buccal swaps (BuccalFix™ Plus DNA Isolation Kit, Isohelix, United Kingdom) as well as other 46 tissue samples following the manufacturer’s instructions (QIAamp Qiagen Mini Kit, cat#51306, Germany). The DNA concentration was assessed using a Qubit 4.0 fluorometer assay (Invitrogen by Thermo Fisher Scientific, Oregon, United States). High-depth, targeted sequencing analysis by amplicon based MPS for the entire mitochondrial DNA was performed for 148 samples (LC Sciences, LLC; VariantPro™ -Mitochondrial Mutations Screening Panel, Houston, TX, United States), achieving median coverage of 7,349 reads. The assay uses 110 primer pairs (VariantPro primer design VariantPro™ primer pools) generating ∼200bp amplicons and providing 100% amplicon coverage of the 16,569 bp of the human mitochondrial genome. Briefly, a total of 100 ng DNA was used for library construction following the manufacturer’s instructions. The sequencing libraries were generated by VariantPro™ protocol (LC Sciences, United States) and sequenced on NovaSeq6000 (Illumina, San Diego, United States) with PE150 (paired-end 150bp). To minimize the risk of allelic dropout during PCR due to primers that are not able to bind, SNPs with a frequency >10% were not allowed in the primer priming region. The VariantPro™ utilizes a nested PCR thermal cycling protocol: including 30 cycles to anneal specific primers. A total of 15 cycles were used for amplicon generation. mtDNA variants with heteroplasmy levels >5% were included in the analysis, whereas variants in positions where common sequencing errors occur due to a homopolymer stretch (m.302–315 and m.3105–3109) were excluded.

### 2.3 Pre-processing data analysis and mtDNA variant annotation

Pooled sequenced samples were demultiplexed using Picard tools (https://broadinstitute.github.io/picard/). Prior to alignment, the low-quality reads (including reads containing sequencing adaptors and nucleotides with mapping and base quality scores lower than 20) were removed. Cleaned, paired-end reads were subsequently produced. Read pairs were aligned to the hg19 reference sequence using the Burrows-Wheeler Aligner, and data were sorted and duplicate-marked using Picard tools. In the second post-alignment processing step, local read realignment was performed to correct for potential alignment errors. The alignments were refined using the Genome Analysis Toolkit (GATK) for localized realignment around indel sites https://software.broadinstitute.org/gatk/documentation/tooldocs/current/org_broadinstitute_gatk_tools_walkers_indels_IndelRealigner.php). Recalibration of base quality scores was also performed using GATK (http://gatkforums.broadinstitute.org/discussion/44/base-quality-score-recalibration-bqsr). Variant calling was done using GATK HaplotypeCaller or UnifiedGenotyper and variant recalibration was performed using a Gaussian mixture model to assign an accurate confidence score to each putative variant call and evaluate new potential variants.

The mtDNA coverage across all samples was measured using bamstats05 (https://github.com/naumenkosa/bioscripts/blob/07df2807ef0de65a5e377635693e6e5ad9225d30/bam/bam.coverage.bamstats05.sh).

Off-target mtDNA reads were also extracted from exome sequence data, previously reported for 93 TSC-associated tumor samples, 4 TSC patient control tissues, and 6 healthy control tissues (median number of mtDNA reads: 115,574; median coverage: 501x) (Giannikou et al., 2016; Martin et al., 2017; Giannikou et al., 2019). For downstream analyses, only samples with minimum read coverage >100 for mtDNA variant calling were selected. The mtDNA variants were called with the bcbio-nextgen pipeline (https://bcbio-nextgen.readthedocs.io/en/latest/).

All mtDNA variant calls were manually reviewed using the Integrative Genomics Viewer (https://software.broadinstitute.org/software/igv/) and artifacts were filtered out. The remaining variants were then annotated by the Variant Effect Predictor (VEP) and ANNOVAR (Yang and Wang, 2015). Amino acid changes for variants in protein genes were predicted according to the human mitochondrial genetic code.

Patient haplotypes were identified using HaploGrep (v.2.2) (https://haplogrep.i-med.ac.at/app/index.html). The HmtVar database was used to further investigate mtDNA variability across different haplogroups and explore the pathogenicity of the mtDNA variants identified across all samples (Preste et al., 2019). Variants were submitted programmatically to the HmtVar API, returning a JSON file for parsing. Disease scores, pathogenicity predictions from several tools and mtDNA databases (e.g., MITOMAP, GnomAD), as well as allele frequency from different ethnic groups across different geographic regions were among the data queried from the HmtVar database. We used MseqDR, Varsome, ClinVar, GnomAD, MITOMAP, and Mitimpact to check these variants for commonality and causality in the general population and other diseases (Lott et al., 2013; Kopanos et al., 2019).

### 2.4 mtDNA copy number analysis

We performed copy number mtDNA analysis using NovaQUANT™ Human Mitochondrial to Nuclear DNA Ratio Kit (Sigma Millipore, Cat. No. 72620) following the manufacturer’s standard instructions. We measured and compared mtDNA copy number to nuclear DNA (nDNA) by quantitative PCR in a set of 35 tumors and 17 corresponding normal tissues.

### 2.5 Statistical analysis

Statistical analyses were performed using GraphPad Prism 8.0 (https://www.graphpad.com/). We applied the Matt-Whitney U test and Kruskal-Wallis test to quantitative variables (two-tailed non-parametric unpaired test) as well as Fisher’s exact test. *p*-values <0.05 were considered statistically significant and the following convention was used: *p ≤ 0.05; **p ≤ 0.01; ***p ≤ 0.001; ****p ≤ 0.0001.

## 3 Results

**FIGURE 1:**
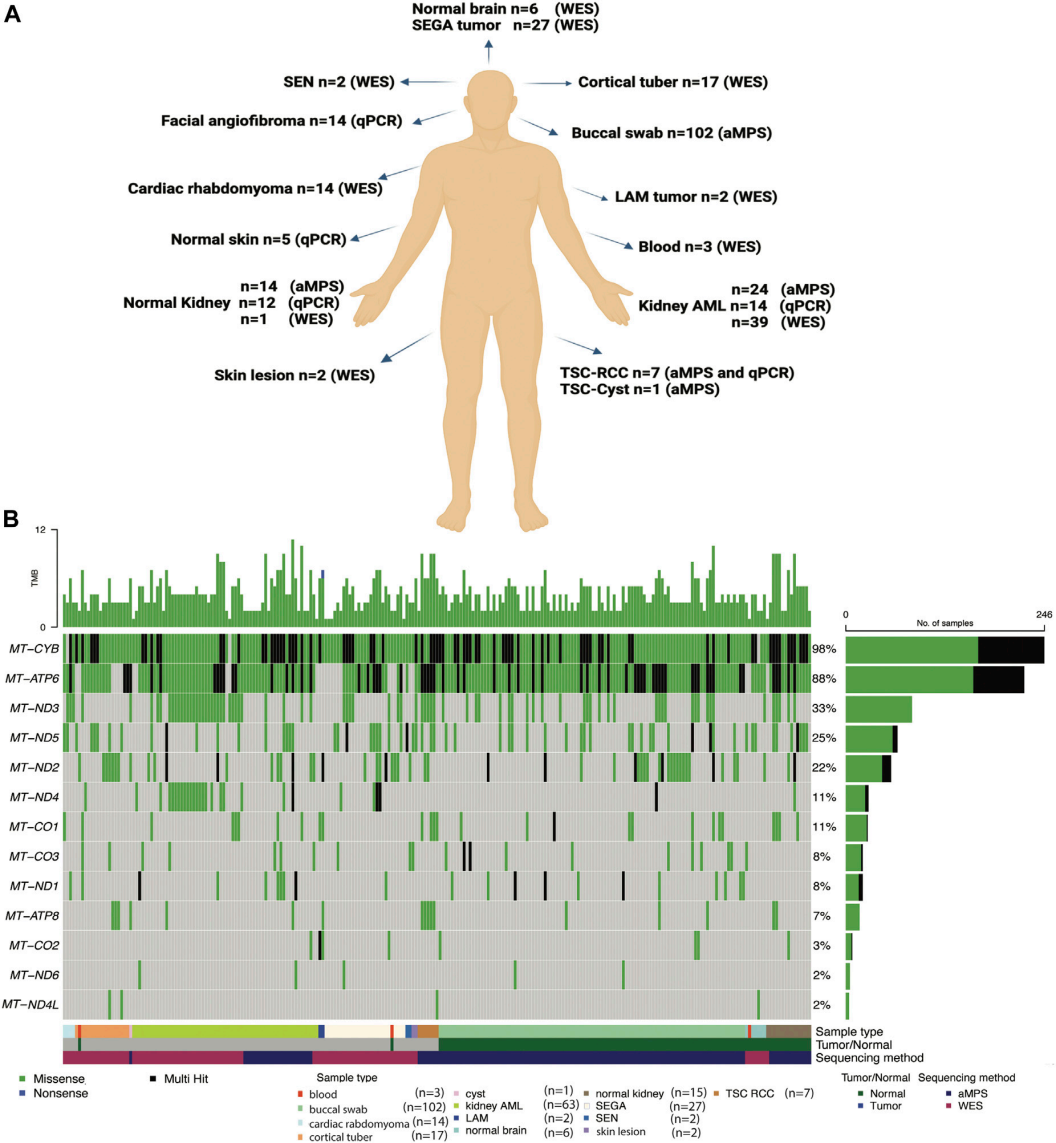
Summary of the TSC samples analyzed, mtDNA analysis methods implemented, and the most common mtDNA variants identified. **(A)** Comprehensive mtDNA analysis was performed in 270 samples (139 tumors and 131 non-tumor tissues) from 199 patients with TSC and 6 healthy controls. The non-tumor tissue samples include 102 buccal swabs. mtDNA was sequenced using amplicon-based massively parallel sequencing (aMPS) and off-target extracted mtDNA reads from whole-exome sequencing (WES) analysis. We also performed mtDNA/gDNA qPCR to assess mitochondrial content. Figure was prepared from a template in Biorender. **(B)** Annotation of the types of mtDNA variants discovered in the 13 protein-coding genes of the mitochondrial electron transport chain in TSC-associated tumors (n = 125) compared to unaffected tissues (n = 126) from 191 patients with TSC and 6 healthy controls. The type of tissue (normal or tumor) and method of mtDNA sequencing is indicated. Missense variants (green), non-sense (blue), and genes with multiple variants identified in a single coding gene (“multi-hit”, black) are indicated in TSC tumors of the skin, blood, kidney, and brain, as well as buccal swabs and normal adjacent tissues.

In this study, we analyzed mtDNA from 139 tumor samples and 131 normal tissue samples from 199 TSC patients and six healthy individuals without TSC (Supplementary Table S1). Our objective was to identify potential germline and somatic disease modifying mtDNA variants. We also determined whether mitochondrial genome copy number is changed in TSC lesions (Figure 1A). To fully leverage available samples and datasets, we combined mtDNA sequencing data from both aMPS and extracted mtDNA off-target reads from prior WES studies (Giannikou et al., 2016; Martin et al., 2017; Giannikou et al., 2019). Samples sequenced with aMPS included: 102 buccal swabs (median coverage 6**,**762x; range: 101-70**,**859x), 24 angiomyolipoma with normal kidney controls (median coverage: 19,560x; range: 223-111,424x), and seven TSC-RCC with one adjacent cyst sample (median coverage: 23,924; range: 105-182,764x). mtDNA sequences extracted from WES (median number of mtDNA reads: 115,574; median coverage: 501x), included: 39 kidney angiomyolipoma as well as two pulmonary LAM (one abdominal lung tumor and one chylous fluid cell cluster sample), 27 subependymal giant cell astrocytomas (SEGAs), 17 cortical tubers, four cardiac rhabdomyoma, and two skin lesions. The most common mtDNA variants in 13 electron transport chain encoding genes across the 251 mtDNA sequenced samples (125 tumors and 126 normal tissue) are presented in Figure 1B. “Multihit” refers to multiple variants in a single gene. There were no instances of multiple SNVs at the same position in the gene for a patient. In Figure 1B we found that *MT-CYB* is highly polymorphic and contains multiple SNVs for many patients and at least one SNV in 99% of all patients, whereas *MT-ND4L* contains the fewest variants in our study population.

### 3.1 Correlation of clinical features to germline mtDNA variants in buccal swab samples

**FIGURE 2:**
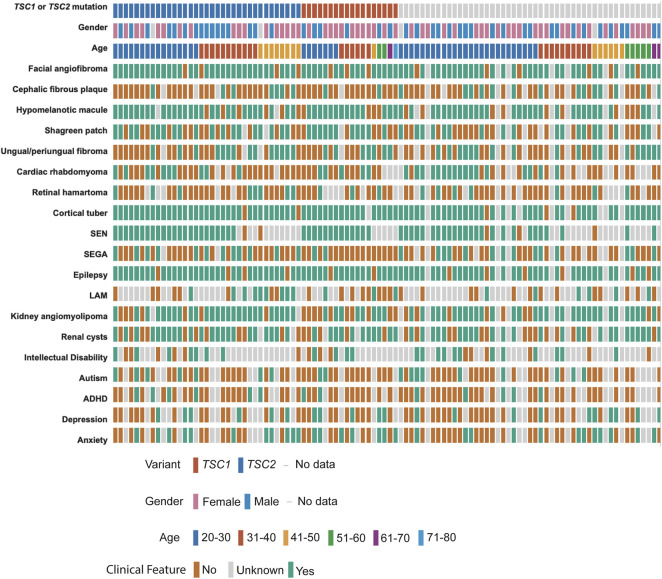
Clinical data of TSC patients with buccal swab samples. Individual patients are shown according to their *TSC1/TSC2* mutation status, gender, and age at the time of buccal swab collection. TSC-pathological features are annotated as present (green), absent (orange), or unknown (grey) according to the information provided by the TSC Alliance Clinical Research Consortium. Figure was prepared in cbioportal https://www.cbioportal.org/oncoprinter.

We performed deep coverage amplicon mtDNA MPS analysis in 102 buccal swab samples (n = 44 male, n = 54 female, median age = 31 years). While all the individuals had a definite TSC diagnosis, detailed clinical information was available for 95/102 cases (Figure 2; Table 1). *TSC1* (18%) and *TSC2* (34%) pathogenic variants were known for 52 cases, while the remainder had unknown mutation status due to either the lack of genetic analysis or no mutation identified after conventional analysis. The number of clinical features was similar between these three groups of patients (Supplementary Figure S1A, Supplementary Table S2).

Five homozygous small indels, two of which were recurrent, with uncertain clinical significance, and variant allele frequency (VAF) > 97% were identified in five individuals with TSC (Supplementary Table S3) (McCormick et al., 2020). These indels primarily affected the mitochondrial non-coding regions NC-5 and NC-7. These five cases did not show any differences regarding the number of clinical features or number of mtDNA single nucleotide variants (SNVs) in comparison to other TSC cases (Supplementary Figure S1B,C).

**FIGURE 3:**
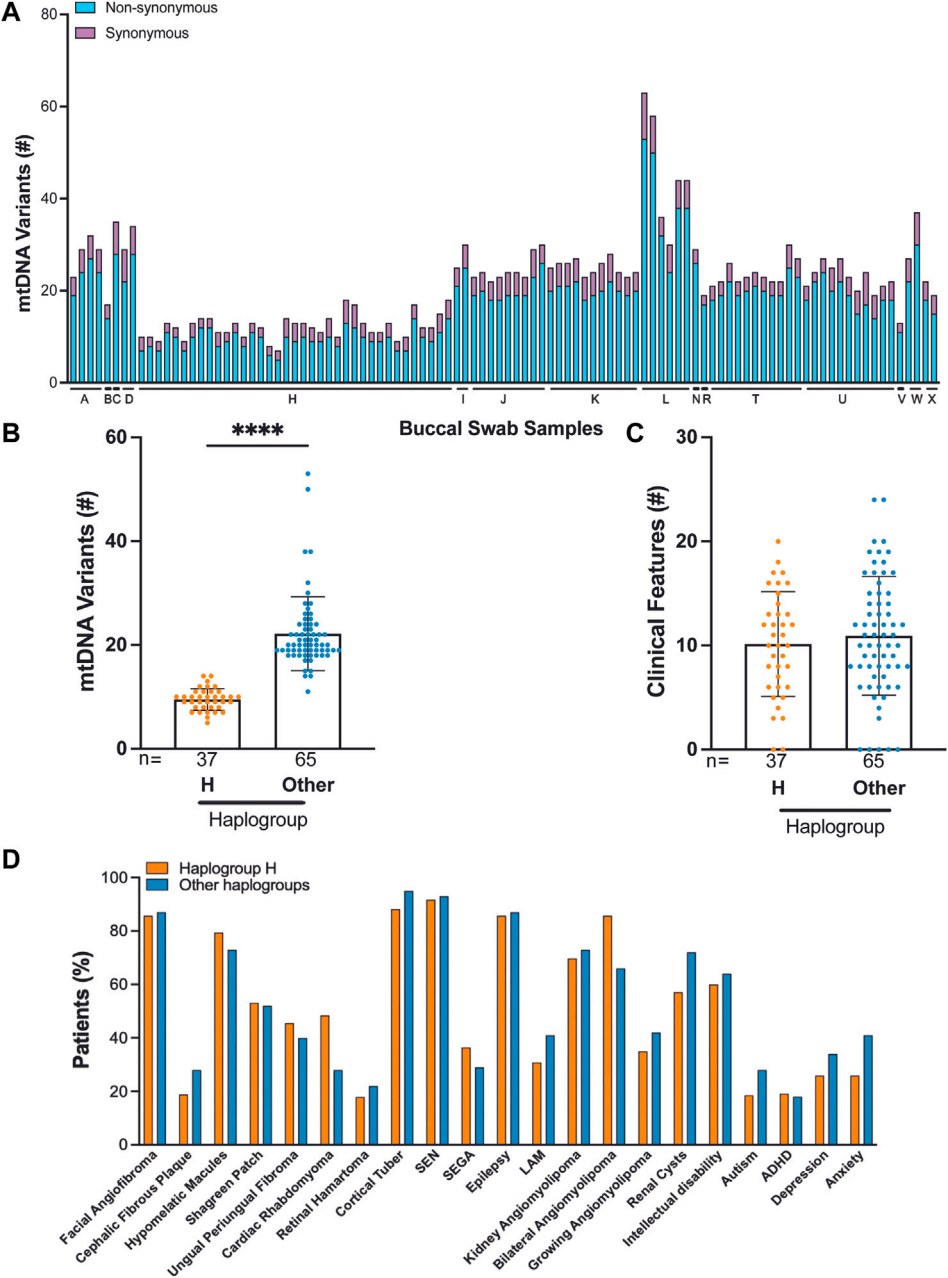
Correlation of TSC-associated clinical features and mtDNA haplogroup analysis from 102 buccal swabs. **(A)** Distribution of synonymous and non-synonymous variants in TSC individuals according to their haplogroup. **(B)** Number of mtDNA variants in haplogroup H compared to all other haplogroups. **(C,D)** The frequency of 22 clinical features in patients from haplogroup H compared to all other haplogroups.

We identified 16 haplogroups in this cohort with multiple haplotypes represented (Supplementary Figure S1D). Samples were differentially distributed across haplogroups: H haplogroup (37/102, 36%), U haplogroup (11/102, 11%), K haplogroup (11/102, 11%), T haplogroup (11/102, 11%), and J haplogroup (9/102, 9%). H haplogroup was the most common in this cohort, reflecting populations of European ancestry or Lineage N (“Eurasian”) (Figure 3A, Supplementary Table S2). We performed mtDNA variant calling using GATK, and we observed that the H haplogroup is overrepresented in the reference genome resulting in an increased number of variant calls in other haplogroups (Figure 3B) (Fatumo et al., 2022a).

Out of 102 buccal swab samples analyzed, we identified 1,791 SNVs, of which 396 were missense, 648 were synonymous, and 747 were non-coding alterations (Supplementary Table S4) (McCormick et al., 2020). This set of 1,791 mtDNA variants included 278 novel alterations. The median burden of non-synonymous SNVs was four per buccal swab sample with a range of 2–10 (Supplementary Table S2). The majority of non-synonymous SNVs, 390 out of 396 (98%), occurred at VAF:>95%, reflecting homoplasmic, germline variants. We found that C>T and T>C single nucleotide substitutions were the most predominant alterations in our cohort consistent with other studies (Supplementary Figure S1E) (Ju et al., 2014; Stewart et al., 2015; Stewart and Chinnery, 2021). We found no significant correlation between mtDNA variants and TSC features, particularly in the comparison of haplogroup H to all other haplogroups in our study (Figures 3C,D). We also observed that the number of mtDNA germline variants in this cohort does not correlate with age and does not significantly vary in patients with either *TSC1* or *TSC2* alterations (Matt Whitney *t*-test) (Supplementary Figures S1F,G). In three families, we identified mtDNA variants consistent with established maternal inheritance, and observed that the clinical significance is not changed between cases of inherited and sporadic TSC (Supplementary Figure S2).

The vast majority of the non-synonymous SNVs 396 identified in buccal swab samples were missense variants of uncertain clinical significance. While *MT-CYB* harbored the greatest and *MT-ND4L* harbored the least amount of genetic variability across the samples (Figure 1B), no pathogenic variants were identified in the buccal swab samples (Supplementary Table S4) (McCormick et al., 2020).

In summary, our analysis revealed common germline mtDNA variants observed in the general population, including the observations that T>C substitutions are the most common in this cohort.

### 3.2 Somatic mtDNA variants in TSC related tumors

**FIGURE 4:**
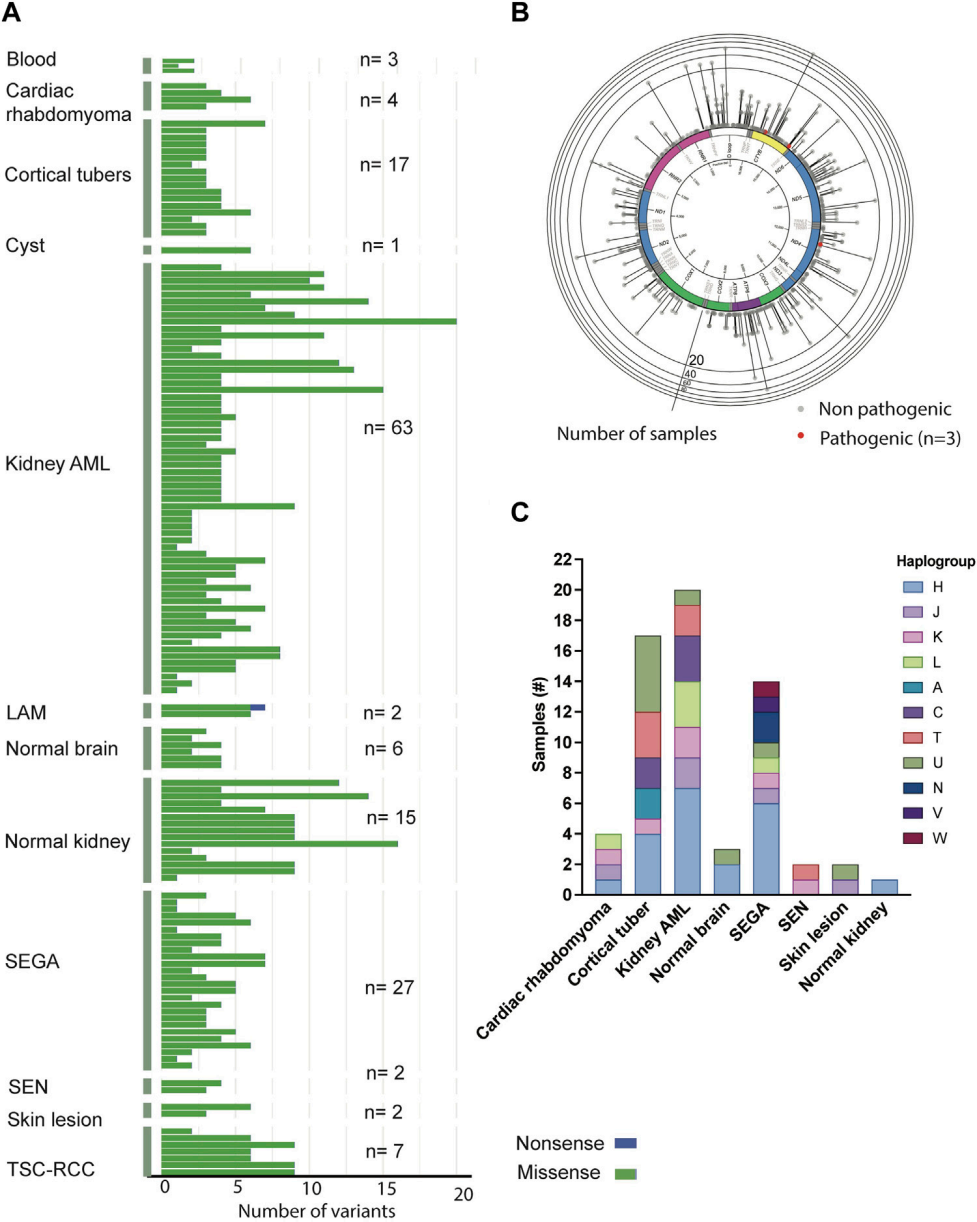
Analysis of mtDNA in TSC-associated tumors. **(A)** Distribution and frequency of missense, non-sense, and frameshift variants identified in TSC-associated tumors and corresponding normal tissues. **(B)** Gene location and prevalence of mtDNA variants identified in TSC-associated tumors. Red spots indicate three potentially pathological mtDNA variants identified by *in silico* prediction software and annotated in Table 2. **(C)** Distribution of haplogroups across 63 TSC-associated tumors (n = 63).

Somatic alterations in the mtDNA have been reported in various cancers including pediatric cancers (Ju et al., 2014; Triska et al., 2019; Yuan et al., 2020), suggesting that mtDNA variants may promote tumorigenesis. To identify mtDNA variants in TSC tumors, we analyzed aMPS and extracted off-target mtDNA reads from WES (Figure 4A, Supplementary Figures S3A–C, Supplementary Tables S5–S11). In 125 TSC-associated tumors, we discovered three predicted pathogenic variants at VAF of 7%–43% in Complex I and Complex III of the electron transport chain (Figures 4A,B; Table 2). There were no large mtDNA deletions or indels found (Supplementary Tables S5, S7). We identified somatic variants using normal adjacent tissue or multiple tumors from one TSC individual (MT-p129 with 16 kidney AML samples) (Table 3).

**TABLE 2:** In silico predicted mtDNA pathogenic variants.

Sample type	Patient ID	Variant	Gene	VAF (%)	Variant Type	Amino acid change
AML (aMPS)	MT-p119	m.11742G>A	*MT-ND4*	43	Missense	p.Cys328Tyr
LAM (WES)	MT-p150	m.14775T>C	*MT-CYB*	43	Missense	p.Leu10Pro
RCC (aMPS)	MT-p196	m.15555C>T	*MT-CYB*	7	Missense	p.Pro270Leu

**TABLE 3:** Somatic mtDNA variants identified in TSC-associated tumor samples.

Sample	Patient ID	Variant	Gene	VAF (%)	Type	Amino acid change
Kidney AML (aMPS)	MT-p197	m.6038C>A	*MT-CO1*	8	Synonymous	p.Gly45Gly
Kidney AML (WES)	MT-p129	m.12561G>A	*MT-ND5*	13	Synonymous	p.Gln75Gln
	MT-p129	m.9445G>A	*MT-CO3*	18	Missense	p.Arg90Gln
	MT-p129	m.1474G>A	*MT-RNR1*	92	Non-coding	—
	MT-p129	m.3163G>A	*MT-RNR2*	69	Non-coding	—
	MT-p129	m.12470T>C	*MT-ND5*	11	Missense	p.lso45Thr
SEGA (WES)	MT-p181	m.11150G>A	*MT-ND4*	13	Missense	p.A1a131Thr
	MT-p186	m.2379C>T	*MT-RNR2*	99	Non-coding	—
RCC (aMPS)	MT-p192	m.10248T>C	*MT-ND3*	19	Synonymous	p.Leu64Leu
	MT-p196	m.2005C>T	*MT-RNR2*	8	Non-coding	—
	MT-p196	m.5979G>A	*MT-CO1*	8	Missense	p.Ala26Thr
	MT-p196	m.10635G>T	*MT-ND4L*	10	Missense	p.Ala56Ser
	MT-p196	m.16161T>C	*MT-CR*	12	Non-coding	—
	MT-p196	m.16478C>A	*MT-CR*	16	Non-coding	—
Cortical Tubers (WES)	MT-p157	m.2259C>T	*MT-RNR2*	100	Non-coding	—

Similar to the buccal swab analysis, haplogroup H was the most prevalent (33%) in our analysis including 63 tissue samples (angiomyolipoma, SEGA, SEN, cortical tubers, cardiac rhabdomyoma, skin lesion, as well as normal kidney and brain) (Figure 4C, Supplementary Table S12). The next largest haplogroup was U including 16% of the samples. While H was the most predominant haplogroup in the 63 samples, 11 other haplogroups were also represented in our cohort (Figure 4C, Supplementary Table S12). Overall, we observe no correlation among any of the clinical features and certain haplogroups.

#### 3.2.1 Kidney angiomyolipoma and lymphangioleiomyomatosis

Approximately 70% of patients with TSC develop multiple bilateral kidney angiomyolipoma, which are unique tumors characterized by abnormal, asymmetric blood vessels, adipocytes, and smooth muscle-like cells (Lam et al., 2018). To characterize the kidney angiomyolipoma mtDNA from patients, we performed aMPS analysis in 24 kidney angiomyolipoma and 11 normal kidneys (Supplementary Tables S5–S7). Normal adjacent kidney tissue was available for eight kidney angiomyolipoma, in which one somatic synonymous variant was identified in a single tumor sample (MT-p197; m.6038C>A, p. Gly45Gly, VAF: 8%, Table 3). This synonymous variant is annotated in MitoMap with a population frequency of 0.002%. Through *in silico* predictions, we also found one *in silico* predicted pathogenic variant in *MT-ND4* (m.11742T>C, p. Cys328Tyr, VAF: 42.5%), which has not been annotated in MitoMap (Figure 4B; Table 2).

In addition, analysis of mtDNA from off-target exome sequencing reads of 39 kidney angiomyolipoma and two pulmonary LAM tumors, a similar tumor entity to kidney angiomyolipoma (median VAF: 99.6%) was performed (Supplementary Figures S3, S8 and Supplementary Table S9). There were also five unique variants within the 16 kidney angiomyolipoma from a single TSC patient (MT-p129) that are included in Table 3. *In silico* prediction and Mitimpact analyses revealed one somatic pathogenic variant in *MT-CYB* of a LAM-associated abdominal tumor (m.14775T>C, p.Leu10Pro, VAF: 42.9%) (Figure 4B; Table 2).

#### 3.2.2 Renal cell carcinoma

Approximately 3% of TSC patients develop renal cell carcinoma (TSC-RCC) (Yang et al., 2014; Henske et al., 2021). To uncover the mitochondrial landscape of TSC-RCC, we performed mtDNA aMPS in 12 kidney samples from six TSC patients that included TSC-RCC with an adjacent cyst and normal kidney. Analysis of the seven TSC-RCC samples and one TSC-cyst revealed six unique somatic variants with low VAF with a range of 8%–19% (Table 3, Supplementary Table S8). *In silico* prediction analysis identified a potentially pathogenic variant in *MT-CYB* (m.15555C>T, p. Pro270Leu, VAF: 7%) (Figure 4B; Table 2). The variant m.15555C>T occurred in *MT-CYB*, the gene harboring the greatest number of SNV across all samples (Figure 1B, Supplementary Table S6).

#### 3.2.3 Cortical tubers and subependymal giant cell astrocytoma

In our study, we also analyzed 17 cortical tubers with off-target mtDNA data from WES (Supplementary Figure S3, Supplementary Table S9). Analysis of two SEGA samples, one SEN, one cortical tuber and two normal brain samples from the same patient (MT-p157) revealed one somatic variant in *MT-RNR2* (m.2259C>T) seen in all tumor samples with variable VAFs (Table 3, Supplementary Table S10). This variant occurs at a frequency of 0.532% reported in MitoMap and as high as 80% in certain haplogroups.

We also analyzed off-target mtDNA reads from high coverage exome sequencing data in 27 SEGA tumors, of which six had matching normal samples. In those six pairs, two somatic variants not seen in other SEGA samples, were identified (m.2379C>T, VAF: 99% and m.11150G>A, VAF: 13%) (Table 3, Supplementary Tables S8–S10). In one SEGA with a corresponding normal brain sample (MT-p181), a somatic missense variant was identified in *MT-ND4* (m.11150G>A, VAF: 13%), which has been described previously (Table 3) (Liu et al., 2001).

### 3.3 Somatic variants identified in nuclear encoded mitochondrial genes

We inquired whether there are any notable alterations in mitochondrial genes encoded by the nuclear genome by filtering the prior analysis of variants published in the WES datasets with MitoCarta 3.0, a comprehensive list of mitochondria-associated genes (Giannikou et al., 2016; Martin et al., 2017; Giannikou et al., 2021; Rath et al., 2021). The majority of variants occured at a low VAF and affected genes involved in mitochondrial metabolism. Two variants in particular play a central role in mitochondrial function: *NDUFA8* is an integral component of Complex I in the electron transport chain and *MRSP23* is a component of the mitochondrial protein translation machinery. In the kidney angiomyolipoma datasets we identified four potentially deleterious variants in nuclear-encoded mitochondrial-associated genes including: ATPase Copper Transporting Beta (*ATP7B*; c.2173A>T, p.Arg725Trp, VAF:40%), *NADH* Ubiquinone Oxidoreductase Subunit A8 (*NDUFA8*; c.284A>G, p.Gln95Arg, VAF: 85%), Glycine C-Acetyltransferase (GCAT; c.1201G>T, p. Gly401Trp, VAF: 89%) and Phosphodiesterase 2A (*PDE2A*; c.930C>A, p. Asp310Glu, VAF: 12%) (Giannikou et al., 2016). In cortical tubers we identified six likely deleterious variants. One tuber (MT-p132) contained a variant in 5′-Nucleotidase Domain Containing 2 (*NT5DC2*; chr3:g.52562492C>A, VAF: 7%). Another tuber which had low mtDNA coverage and was not part of our mitochondrial genome analysis contained five variants predicted to be deleterious: Mitochondrial Small Ribosomal Subunit Protein 23 (MRPS23; chr17:g.55926600C>A, VAF:8%), Acyl-CoA Synthetase Family Member 2 (*ACSF2*; chr17:g.48540546C>A, VAF:15%), Pyruvate Dehydrogenase Phosphatase Regulatory Subunit (*PDPR*; chr16:g.70165250C>A, VAF: 4%), Solute Carrier Family 25 Member 48 (*SLC25A48*; chr5:g.135188365G>T, VAF: 9%), and Aldehyde Dehydrogenase 1 Family Member L1 (*ALDH1L1*; chr3:g.125844487C>A, VAF: 15%) (Supplementary Table S13 (Martin et al., 2017)). Interestingly, there were no variants identified in well-known quality control proteins such as PINK1 or PARKIN that might affect with removal of damaged mitochondria by mitophagy.

### 3.4 mtDNA copy number analysis in TSC related tumors

**FIGURE 5:**
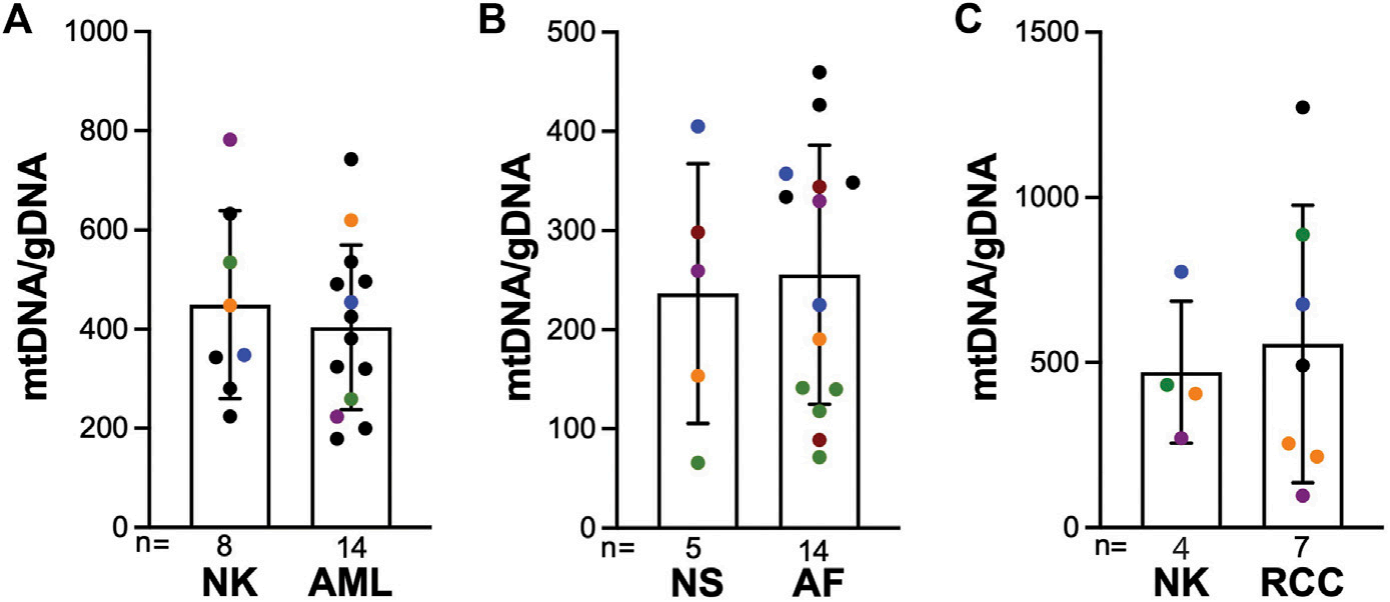
mtDNA/gDNA is unchanged in TSC tumors compared to corresponding normal tissue. **(A–C)** mtDNA/gDNA was measured in renal angiomyolipoma (AML), angiofibroma (AF), and renal cell carcinoma (RCC) compared to normal kidney (NK) or normal skin (NS). Black dots are unmatched samples, and all other colors indicate matched tumor-normal samples within each analyzed tissue.

mtDNA abundance varies significantly across tissues and tumor types. Notably, mtDNA/gDNA levels are increased in preclinical neuronal models of TSC (Goto et al., 2011; Ebrahimi-Fakhari et al., 2016; Yuan et al., 2020). Copy number analysis by qPCR did not reveal significant changes in mitochondrial content (mtDNA/gDNA) in kidney angiomyolipoma, facial angiofibroma, or TSC-RCCs compared to corresponding normal tissues (Figure 5, Supplementary Tables S4, S5, S8, S14). In particular, we did not observe any consistent correlation between the mtDNA copy numbers in 19 TSC-associated tumors (four angiomyolipomas, ten angiofibromas, five TSC-RCC including a cyst adjacent to tumor) compared to the mtDNA copy number in matching normal tissue. These data further demonstrate the relative stability of the mtDNA genome in TSC tumors, consistent with their benign nature and relatively low mitotic index.

## 4 Discussion

TSC is characterized by broad phenotypic heterogeneity, suggesting that other events beyond *TSC1/TSC2* biallelic loss may act as disease modifiers and susceptibility loci for tumor development (Henske et al., 2016; Martin et al., 2017). Herein, we performed mtDNA genetic analysis of 270 samples from 199 TSC patients and six healthy controls using high throughput sensitive variant detection methods, including amplicon MPS and WES. We defined the spectrum of mtDNA germline and somatic variant burden and correlate it with clinical features. We observed variability in the number of mutant mitochondrial genes and mtDNA variants across different TSC samples. Consistent with prior mtDNA analysis of 1,675 different tumors from diverse human cancer types, we also found that C>T and T>C transitions are the most prevalent alterations in TSC tissues (Ju et al., 2014; Stewart et al., 2015; Stewart and Chinnery, 2021). This is the first comprehensive deep coverage mtDNA genetic analysis in the largest cohort ever enrolled for patients with TSC. While the prevalence of TSC is independent of race (Henske et al., 2016), this is the first study to provide initial evidence that the clinical manifestations of TSC may not be significantly affected by race.

We identified three variants of uncertain significance (VUS) predicted to be pathogenic by *in silico* algorithms, one in *MT-ND4* and two in *MT-CYB* occurring in a kidney angiomyolipoma, a TSC-RCC, and a LAM-associated abdominal tumor. There were no pathogenic variants identified in the buccal swab samples. Due to the diversity of tumor types, various analyses, and repetitive sampling from the same individual, this study is complex. As mitochondria can vary in a cell, tissue, and context-specific manner and given the variability of the samples analyzed, it is not surprising that recurrent somatic changes were not found in this study. Future approaches including data from larger populations may facilitate variant annotation and data interpretation, thereby allowing better correlation of genotype-phenotype. Similarly, studies in Fabry Disease and prostate cancer emphasized the need for large and diverse sample size in mtDNA studies as well (Simoncini et al., 2016; Kalsbeek et al., 2017; Schopf et al., 2020). Further analysis of additional samples in larger cohorts and functional studies in preclinical models are required to clarify the potential TSC disease-modifying implications of the mtDNA variants identified in our study.

Identification of disease modifying variants is particularly challenging as a result of heteroplasmy and the highly polymorphic nature of mitochondrial genome (Wang et al., 2012). Previous studies have shown that mtDNA variation could contribute to the differences in disease prevalence observed among different ethnic groups (Calvo et al., 2006; Tasdogan et al., 2020; Crooks et al., 2021; Orsucci et al., 2021). Certain mtDNA variants can be pathogenic in individuals of certain ancestry and haplotype or occur more frequently in a specific haplogroup (Wei et al., 2017). In addition, other variants are polymorphic and commonly observed in different mitochondrial lineages with different haplogroup backgrounds. Some variants are haplogroup-defining and rarely found in other populations. Finally, there are variants that are haplogroup-specific and polymorphic at the same time (Falk et al., 2015). Importantly, the clinical significance of variants may be further complicated by instances in which the pathogenicity is affected by the overall mitochondrial haplogroup (Kim et al., 2008; Falk et al., 2015; Tasdogan et al., 2020). Common complex diseases have been associated with specific mitochondrial haplogroups, including Alzheimer’s disease with haplogroup U (van der Walt et al., 2004) and age-related macular degeneration with the JTU haplogroup cluster (Kenney et al., 2013). In this cohort, we did not find any pathogenic or likely pathogenic mtDNA variants previously associated with any specific haplogroup.

This study has a few notable limitations including a relatively small sample size from each tissue type and haplogroup. Furthermore, the genetic status of *TSC1* and *TSC2* was only known for about half of the patients providing buccal swab samples. The study lacked adequate sampling of these two patient populations to observe the well-established clinical association of *TSC2* alterations with more severe disease burden compared to patients with *TSC1* alterations. Also, there is no correlation between particular *TSC1 or TSC2* genetic variants with certain phenotypes/clinical features. Taking into consideration the phenotypic complexity of the disease, the same TSC clinical features (e.g., intellectual disability, autism) can be associated with a broad spectrum of phenotypic heterogeneity (mild to severe) across different patients who harbor the same genetic alterations, making feature classification challenging.

In our analysis more variants were identified in individuals outside of the European ancestry suggesting that haplogroup specific mtDNA reference genomes may be beneficial as genomics studies focus on important diversity initiatives (Fatumo et al., 2022a; Fatumo et al., 2022b). mtDNA analysis in larger cohorts including more tumor and corresponding normal tissue pairs would significantly improve our understanding of the acquisition of somatic variants in the mtDNA of TSC tumors. Furthermore, analysis of discordant monozygotic twins affected by TSC may also enable a better understanding of the connection between mitochondrial variants and heterogeneity in TSC clinical features. The purity of the tumor samples was not assessed in this study. The overall mtDNA analysis in tumors may be affected by mixture with adjacent normal cells and other infiltrating stromal and immune cell populations. This may be particularly relevant in facial angiofibromas from circular 2-mm punch biopsies (Klonowska et al., 2022).

Taken together, our data demonstrate the high stability of the mitochondrial genome in various TSC lesions suggesting that the acquisition of pathogenic mtDNA variants is a rare occurrence in tumors associated with TSC that may reflect the expected acquisition of somatic variants associated with cell proliferation (Ju et al., 2014; Stewart et al., 2015; Stewart and Chinnery, 2021). We identified several somatic likely pathogenic mtDNA variants across different TSC tissues as wells as essential nuclear encoded mitochondrial genes, which warrant further investigation within the context of TSC pathogenesis (Supplementary Table S13). Future studies should focus on the effect of mitochondrial genetic variation on specific phenotypes and analysis of nuclear-mitochondrial genome interactions to characterize the molecular pathways driven by these mitochondrial alterations, define the clinical implication of likely pathogenic mtDNA variants, and address the therapeutic potential for targeting mutation-induced metabolic dysregulation in TSC related tumors. Such studies will help uncover the contribution of mitochondrial genetic variation to TSC, other complex diseases as well as normal trait variance.

## Data Availability

The data presented in the study are deposited in the NCBI SAR repository, accession number PRJNA910832.

## Supplementary material

SUPPLEMENTARY FIGURE S1 Correlation of mtDNA variants with age and clinical features in buccal swab samples. **(A)** Twenty-two clinical features were equally prevalent in patients with *TSC1*, *TSC2,* or unknown causative variants. **(B)** Twenty-two clinical features were evenly distributed across patients with mtDNA indels (n=5) compared to patients without indels (n=97). **(C)** The overall number of mtDNA variants is not different in cases with indels (n=5) compared to samples without indels (n=97). **(D)** Individual representation of haplogroups for each patient and the number of mtDNA variants. **(E)** The primary mtDNA variants identified in buccal swab samples were T>C and C>T substitutions. **(F)**, **(G)** The number of mtDNA variants identified in buccal swabs was not significantly affected by the age or TSC mutation status.

SUPPLEMENTARY FIGURE S2 Analysis of mtDNA variants in familial TSC versus sporadic TSC in buccal swabs. **(A)** Family trees from two familial cases and one duo (offspring). These figures highlight the maternal germline inheritance pattern of mtDNA variants in parents and siblings. Black scheme: affected probands, grey: probands with unknown status, white: healthy individual. Numbers represent the number of mtDNA variants identified in every individual. **(B)**, **(C)** The number of mtDNA variants and clinical features in buccal swabs was not significantly affected by familial TSC.

SUPPLEMENTARY FIGURE S3 Summary of mtDNA alterations in tumor and normal tissues. **(A)** Mean number of single nucleotide variants per tissue type in 125 tumor samples and 24 corresponding normal samples distributed by missense, nonsense, frameshift variants. **(B)** Presentation of the top mutated genes and the most frequent nucleotide substitution for each tissue type. **(C)** Mean number of indels per tissue type in 125 tumor samples and 24 normal samples.

## References

[B1] Bessis D. Malinge M. C. Girard C. (2018). Isolated and unilateral facial angiofibromas revealing a type 1 segmental postzygotic mosaicism of tuberous sclerosis complex with c.4949_4982del TSC2 mutation. Br. J. Dermatol. 178 (1), e53–e54. 10.1111/bjd.15868 29315486

[B2] Calvo S. Jain M. Xie X. Sheth S. A. Chang B. Goldberger O. A. (2006). Systematic identification of human mitochondrial disease genes through integrative genomics. Nat. Genet. 38 (5), 576–582. 10.1038/ng1776 16582907

[B3] Crooks D. R. Maio N. Lang M. Ricketts C. J. Vocke C. D. Gurram S. (2021). Mitochondrial DNA alterations underlie an irreversible shift to aerobic glycolysis in fumarate hydratase-deficient renal cancer. Sci. Signal. 14 (664), eabc4436. 10.1126/scisignal.abc4436 33402335 PMC8039187

[B4] de Matos M. R. Posa I. Carvalho F. S. Morais V. A. Grosso A. R. de Almeida S. F. (2019). A systematic pan-cancer analysis of genetic heterogeneity reveals associations with epigenetic modifiers. Cancers (Basel) 11 (3), 391. 10.3390/cancers11030391 30897760 PMC6468518

[B5] Drusian L. Nigro E. A. Mannella V. Pagliarini R. Pema M. Costa A. S. H. (2018). mTORC1 upregulation leads to accumulation of the oncometabolite fumarate in a mouse model of renal cell carcinoma. Cell Rep. 24 (5), 1093–1104. 10.1016/j.celrep.2018.06.106 30067967

[B6] Duvel K. Yecies J. L. Menon S. Raman P. Lipovsky A. I. Souza A. L. (2010). Activation of a metabolic gene regulatory network downstream of mTOR complex 1. Mol. Cell 39 (2), 171–183. 10.1016/j.molcel.2010.06.022 20670887 PMC2946786

[B7] Ebrahimi-Fakhari D. Saffari A. Wahlster L. Di Nardo A. Turner D. Lewis T. L. Jr. (2016). Impaired mitochondrial dynamics and mitophagy in neuronal models of tuberous sclerosis complex. Cell Rep. 17 (4), 1053–1070. 10.1016/j.celrep.2016.09.054 27760312 PMC5078873

[B8] Falk M. J. Shen L. Gonzalez M. Leipzig J. Lott M. T. Stassen A. P. (2015). Mitochondrial disease sequence data resource (MSeqDR): A global grass-roots consortium to facilitate deposition, curation, annotation, and integrated analysis of genomic data for the mitochondrial disease clinical and research communities. Mol. Genet. Metab. 114 (3), 388–396. 10.1016/j.ymgme.2014.11.016 25542617 PMC4512182

[B9] Fatumo S. Chikowore T. Choudhury A. Ayub M. Martin A. R. Kuchenbaecker K. (2022). A roadmap to increase diversity in genomic studies. Nat. Med. 28, 243–250. 10.1038/s41591-021-01672-4 35145307 PMC7614889

[B10] Fatumo S. Yakubu A. Oyedele O. Popoola J. Attipoe D. A. Eze-Echesi G. (2022). Promoting the genomic revolution in africa through the Nigerian 100K genome project. Nat. Genet. 54 (5), 531–536. 10.1038/s41588-022-01071-6 35534563

[B11] Fontana G. A. Gahlon H. L. (2020). Mechanisms of replication and repair in mitochondrial DNA deletion formation. Nucleic Acids Res. 48 (20), 11244–11258. 10.1093/nar/gkaa804 33021629 PMC7672454

[B12] Giannikou K. Lasseter K. D. Grevelink J. M. Tyburczy M. E. Dies K. A. Zhu Z. (2019). Low-level mosaicism in tuberous sclerosis complex: Prevalence, clinical features, and risk of disease transmission. Genet. Med. 21 (11), 2639–2643. 10.1038/s41436-019-0562-6 31160751

[B13] Giannikou K. Malinowska I. A. Pugh T. J. Yan R. Tseng Y. Y. Oh C. (2016). Whole exome sequencing identifies TSC1/TSC2 biallelic loss as the primary and sufficient driver event for renal angiomyolipoma development. PLoS Genet. 12 (8), e1006242. 10.1371/journal.pgen.1006242 27494029 PMC4975391

[B14] Giannikou K. Zhu Z. Kim J. Winden K. D. Tyburczy M. E. Marron D. (2021). Subependymal giant cell astrocytomas are characterized by mTORC1 hyperactivation, a very low somatic mutation rate, and a unique gene expression profile. Mod. Pathol. 34 (2), 264–279. 10.1038/s41379-020-00659-9 33051600 PMC9361192

[B15] Gorelick A. N. Kim M. Chatila W. K. La K. Hakimi A. A. Berger M. F. (2021). Respiratory complex and tissue lineage drive recurrent mutations in tumour mtDNA. Nat. Metab. 3 (4), 558–570. 10.1038/s42255-021-00378-8 33833465 PMC9304985

[B16] Goto J. Talos D. M. Klein P. Qin W. Chekaluk Y. I. Anderl S. (2011). Regulable neural progenitor-specific Tsc1 loss yields giant cells with organellar dysfunction in a model of tuberous sclerosis complex. Proc. Natl. Acad. Sci. U. S. A. 108 (45), E1070–E1079. 10.1073/pnas.1106454108 22025691 PMC3214999

[B17] Henske E. P. Cornejo K. M. Wu C. L. (2021). Renal cell carcinoma in tuberous sclerosis complex. Genes (Basel) 12 (10), 1585. 10.3390/genes12101585 34680979 PMC8535193

[B18] Henske E. P. Jozwiak S. Kingswood J. C. Sampson J. R. Thiele E. A. (2016). Tuberous sclerosis complex. Nat. Rev. Dis. Prim. 2, 16035. 10.1038/nrdp.2016.35 27226234

[B19] Jones A. C. Daniells C. E. Snell R. G. Tachataki M. Idziaszczyk S. A. Krawczak M. (1997). Molecular genetic and phenotypic analysis reveals differences between TSC1 and TSC2 associated familial and sporadic tuberous sclerosis. Hum. Mol. Genet. 6 (12), 2155–2161. 10.1093/hmg/6.12.2155 9328481

[B20] Ju Y. S. Alexandrov L. B. Gerstung M. Martincorena I. Nik-Zainal S. Ramakrishna M. (2014). Origins and functional consequences of somatic mitochondrial DNA mutations in human cancer. Elife 3, e02935. 10.7554/eLife.02935 25271376 PMC4371858

[B21] Kalsbeek A. M. F. Chan E. K. F. Corcoran N. M. Hovens C. M. Hayes V. M. (2017). Mitochondrial genome variation and prostate cancer: A review of the mutational landscape and application to clinical management. Oncotarget 8 (41), 71342–71357. 10.18632/oncotarget.19926 29050365 PMC5642640

[B22] Kenney M. C. Hertzog D. Chak G. Atilano S. R. Khatibi N. Soe K. (2013). Mitochondrial DNA haplogroups confer differences in risk for age-related macular degeneration: A case control study. BMC Med. Genet. 14, 4. 10.1186/1471-2350-14-4 23302509 PMC3566905

[B23] Kim W. Yoo T. K. Shin D. J. Rho H. W. Jin H. J. Kim E. T. (2008). Mitochondrial DNA haplogroup analysis reveals no association between the common genetic lineages and prostate cancer in the Korean population. PLoS One 3 (5), e2211. 10.1371/journal.pone.0002211 18493608 PMC2376063

[B24] Klonowska K. Grevelink J. M. Giannikou K. Ogorek B. A. Herbert Z. T. Thorner A. R. (2022). Ultrasensitive profiling of UV-induced mutations identifies thousands of subclinical facial tumors in tuberous sclerosis complex. J. Clin. Invest. 132 (10), e155858. 10.1172/JCI155858 35358092 PMC9106361

[B25] Kopanos C. Tsiolkas V. Kouris A. Chapple C. E. Albarca Aguilera M. Meyer R. (2019). VarSome: The human genomic variant search engine. Bioinformatics 35 (11), 1978–1980. 10.1093/bioinformatics/bty897 30376034 PMC6546127

[B26] Lam H. C. Siroky B. J. Henske E. P. (2018). Renal disease in tuberous sclerosis complex: Pathogenesis and therapy. Nat. Rev. Nephrol. 14 (11), 704–716. 10.1038/s41581-018-0059-6 30232410

[B27] Liu V. W. Shi H. H. Cheung A. N. Chiu P. M. Leung T. W. Nagley P. (2001). High incidence of somatic mitochondrial DNA mutations in human ovarian carcinomas. Cancer Res. 61 (16), 5998–6001. 11507041

[B28] Lott M. T. Leipzig J. N. Derbeneva O. Xie H. M. Chalkia D. Sarmady M. (2013). mtDNA variation and analysis using mitomap and mitomaster. Curr. Protoc. Bioinforma. 44, 1 23 1–26. 10.1002/0471250953.bi0123s44 PMC4257604 25489354

[B29] Manzanilla-Romero H. H. Weis D. Schnaiter S. Rudnik-Schoneborn S. (2021). Low-level mosaicism in tuberous sclerosis complex in four unrelated patients: Comparison of clinical characteristics and diagnostic pathways. Am. J. Med. Genet. A 185 (12), 3851–3858. 10.1002/ajmg.a.62433 34328706 PMC9291125

[B30] Martin K. R. Zhou W. Bowman M. J. Shih J. Au K. S. Dittenhafer-Reed K. E. (2017). The genomic landscape of tuberous sclerosis complex. Nat. Commun. 8, 15816. 10.1038/ncomms15816 28643795 PMC5481739

[B31] McCormick E. M. Lott M. T. Dulik M. C. Shen L. Attimonelli M. Vitale O. (2020). Specifications of the ACMG/AMP standards and guidelines for mitochondrial DNA variant interpretation. Hum. Mutat. 41 (12), 2028–2057. 10.1002/humu.24107 32906214 PMC7717623

[B32] Northrup H. Aronow M. E. Bebin E. M. Bissler J. Darling T. N. de Vries P. J. (2021). Updated international tuberous sclerosis complex diagnostic criteria and surveillance and management recommendations. Pediatr. Neurol. 123, 50–66. 10.1016/j.pediatrneurol.2021.07.011 34399110

[B33] Orsucci D. Caldarazzo Ienco E. Rossi A. Siciliano G. Mancuso M. (2021). Mitochondrial syndromes revisited. J. Clin. Med. 10 (6), 1249. 10.3390/jcm10061249 33802970 PMC8002645

[B34] Peron A. Au K. S. Northrup H. (2018). Genetics, genomics, and genotype-phenotype correlations of TSC: Insights for clinical practice. Am. J. Med. Genet. C Semin. Med. Genet. 178 (3), 281–290. 10.1002/ajmg.c.31651 30255984

[B35] Preste R. Vitale O. Clima R. Gasparre G. Attimonelli M. (2019). HmtVar: A new resource for human mitochondrial variations and pathogenicity data. Nucleic Acids Res. 47 (D1), D1202–D1210. 10.1093/nar/gky1024 30371888 PMC6323908

[B36] Rath S. Sharma R. Gupta R. Ast T. Chan C. Durham T. J. (2021). MitoCarta3.0: An updated mitochondrial proteome now with sub-organelle localization and pathway annotations. Nucleic Acids Res. 49 (D1), D1541–D1547. 10.1093/nar/gkaa1011 33174596 PMC7778944

[B37] Schopf B. Weissensteiner H. Schafer G. Fazzini F. Charoentong P. Naschberger A. (2020). OXPHOS remodeling in high-grade prostate cancer involves mtDNA mutations and increased succinate oxidation. Nat. Commun. 11 (1), 1487. 10.1038/s41467-020-15237-5 32198407 PMC7083862

[B38] Simoncini C. Chico L. Concolino D. Sestito S. Fancellu L. Boadu W. (2016). Mitochondrial DNA haplogroups may influence Fabry disease phenotype. Neurosci. Lett. 629, 58–61. 10.1016/j.neulet.2016.06.051 27365132

[B39] Stewart J. B. Alaei-Mahabadi B. Sabarinathan R. Samuelsson T. Gorodkin J. Gustafsson C. M. (2015). Simultaneous DNA and RNA mapping of somatic mitochondrial mutations across diverse human cancers. PLoS Genet. 11 (6), e1005333. 10.1371/journal.pgen.1005333 26125550 PMC4488357

[B40] Stewart J. B. Chinnery P. F. (2021). Extreme heterogeneity of human mitochondrial DNA from organelles to populations. Nat. Rev. Genet. 22 (2), 106–118. 10.1038/s41576-020-00284-x 32989265

[B41] Tasdogan A. McFadden D. G. Mishra P. (2020). Mitochondrial DNA haplotypes as genetic modifiers of cancer. Trends Cancer 6 (12), 1044–1058. 10.1016/j.trecan.2020.08.004 32980320

[B42] Taylor R. W. Turnbull D. M. (2005). Mitochondrial DNA mutations in human disease. Nat. Rev. Genet. 6 (5), 389–402. 10.1038/nrg1606 15861210 PMC1762815

[B43] Triska P. Kaneva K. Merkurjev D. Sohail N. Falk M. J. Triche T. J. Jr. (2019). Landscape of germline and somatic mitochondrial DNA mutations in pediatric malignancies. Cancer Res. 79 (7), 1318–1330. 10.1158/0008-5472.CAN-18-2220 30709931 PMC6445760

[B44] Tuppen H. A. Blakely E. L. Turnbull D. M. Taylor R. W. (2010). Mitochondrial DNA mutations and human disease. Biochim. Biophys. Acta 1797 (2), 113–128. 10.1016/j.bbabio.2009.09.005 19761752

[B45] Tyburczy M. E. Dies K. A. Glass J. Camposano S. Chekaluk Y. Thorner A. R. (2015). Mosaic and intronic mutations in TSC1/TSC2 explain the majority of TSC patients with No mutation identified by conventional testing. PLoS Genet. 11 (11), e1005637. 10.1371/journal.pgen.1005637 26540169 PMC4634999

[B46] Tyburczy M. E. Jozwiak S. Malinowska I. A. Chekaluk Y. Pugh T. J. Wu C. L. (2015). A shower of second hit events as the cause of multifocal renal cell carcinoma in tuberous sclerosis complex. Hum. Mol. Genet. 24 (7), 1836–1842. 10.1093/hmg/ddu597 25432535 PMC4355019

[B47] van der Walt J. M. Dementieva Y. A. Martin E. R. Scott W. K. Nicodemus K. K. Kroner C. C. (2004). Analysis of European mitochondrial haplogroups with Alzheimer disease risk. Neurosci. Lett. 365 (1), 28–32. 10.1016/j.neulet.2004.04.051 15234467

[B48] Verhoef S. Bakker L. Tempelaars A. M. Hesseling-Janssen A. L. Mazurczak T. Jozwiak S. (1999). High rate of mosaicism in tuberous sclerosis complex. Am. J. Hum. Genet. 64 (6), 1632–1637. 10.1086/302412 10330349 PMC1377905

[B49] Wang J. Schmitt E. S. Landsverk M. L. Zhang V. W. Li F. Y. Graham B. H. (2012). An integrated approach for classifying mitochondrial DNA variants: One clinical diagnostic laboratory's experience. Genet. Med. 14 (6), 620–626. 10.1038/gim.2012.4 22402757

[B50] Wei W. Gomez-Duran A. Hudson G. Chinnery P. F. (2017). Background sequence characteristics influence the occurrence and severity of disease-causing mtDNA mutations. PLoS Genet. 13 (12), e1007126. 10.1371/journal.pgen.1007126 29253894 PMC5757940

[B51] Yang H. Wang K. (2015). Genomic variant annotation and prioritization with ANNOVAR and wANNOVAR. Nat. Protoc. 10 (10), 1556–1566. 10.1038/nprot.2015.105 26379229 PMC4718734

[B52] Yang P. Cornejo K. M. Sadow P. M. Cheng L. Wang M. Xiao Y. (2014). Renal cell carcinoma in tuberous sclerosis complex. Am. J. Surg. Pathol. 38 (7), 895–909. 10.1097/PAS.0000000000000237 24832166 PMC4139167

[B53] Yu K. H. Miron O. Palmer N. Lemos D. R. Fox K. Kou S. C. (2018). Data-driven analyses revealed the comorbidity landscape of tuberous sclerosis complex. Neurology 91 (21), 974–976. 10.1212/WNL.0000000000006546 30333165 PMC6260203

[B54] Yuan Y. Ju Y. S. Kim Y. Li J. Wang Y. Yoon C. J. (2020). Comprehensive molecular characterization of mitochondrial genomes in human cancers. Nat. Genet. 52 (3), 342–352. 10.1038/s41588-019-0557-x 32024997 PMC7058535

[B55] Zinovkina L. A. (2018). Mechanisms of mitochondrial DNA repair in mammals. Biochemistry. 83 (3), 233–249. 10.1134/S0006297918030045 29625543

