# Supplementary figures and images for: Spectrum of germline and somatic mitochondrial DNA variants in Tuberous Sclerosis Complex
Source: Front Genet. 2023 Jan 30;13:917993. doi: 10.3389/fgene.2022.917993 (PMC9923026; doi:10.3389/fgene.2022.917993)

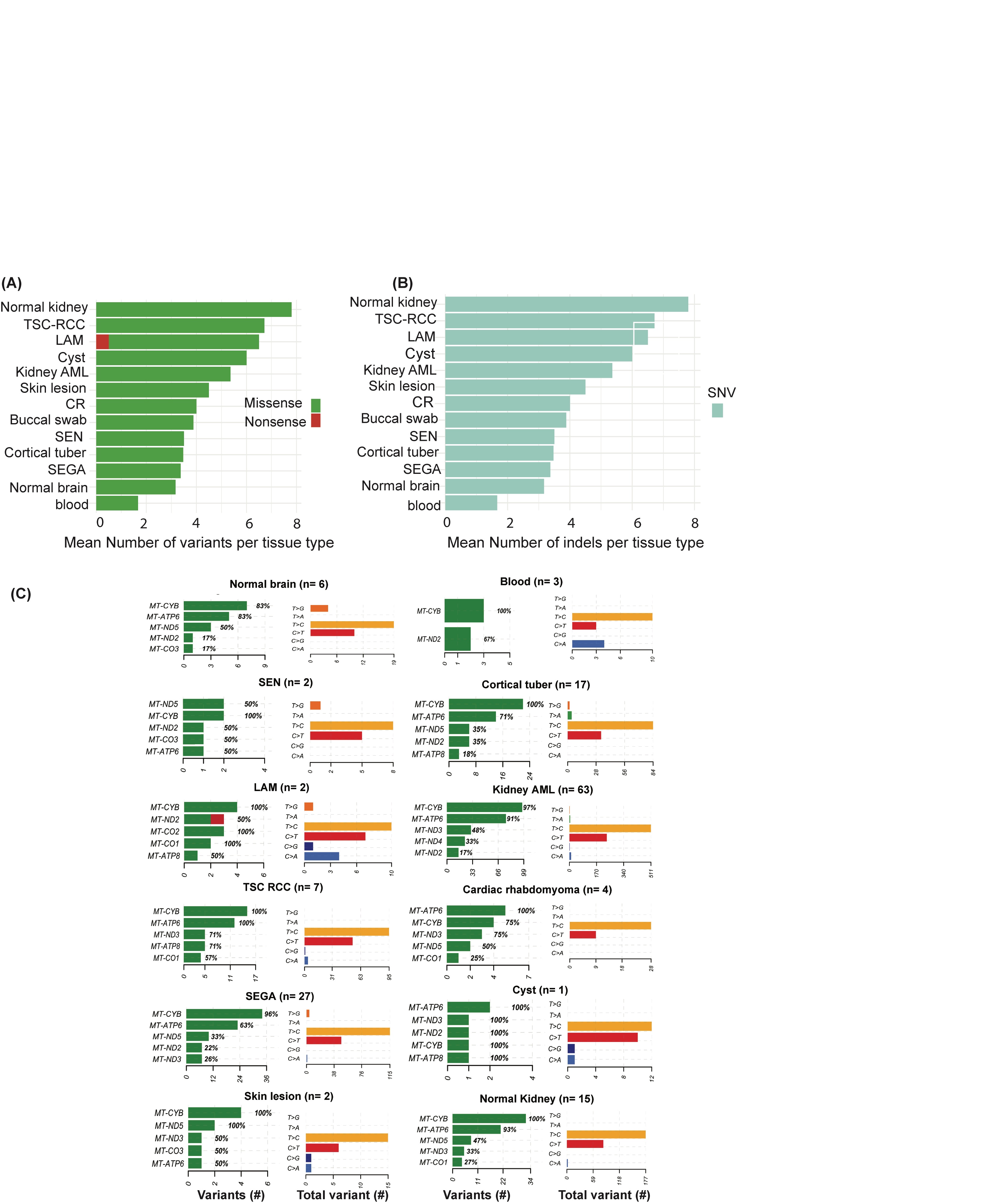

Supplement: Supplementary file 1 [file Image3.jpeg]

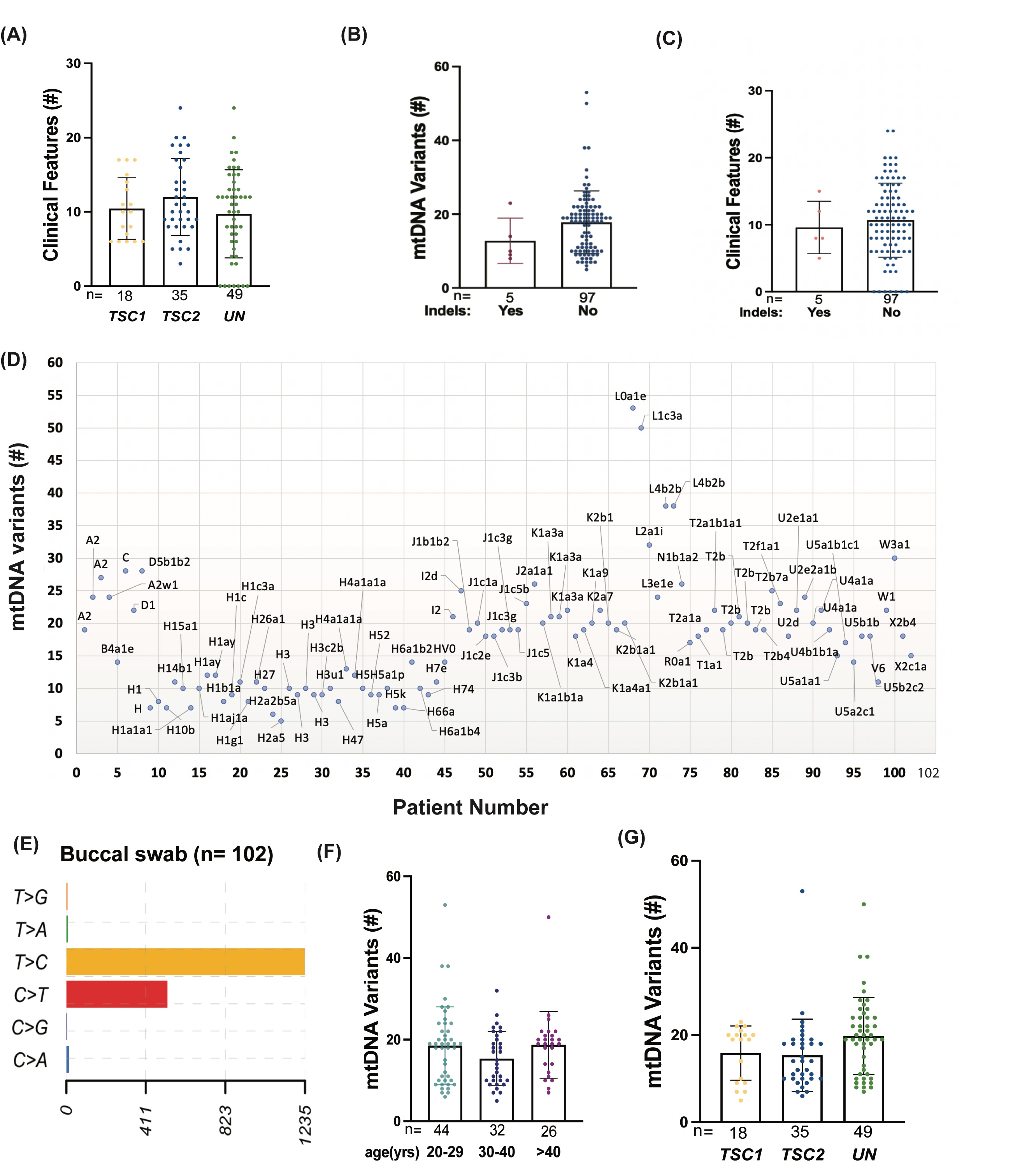

Supplement: Supplementary file 2 [file Image1.jpeg]

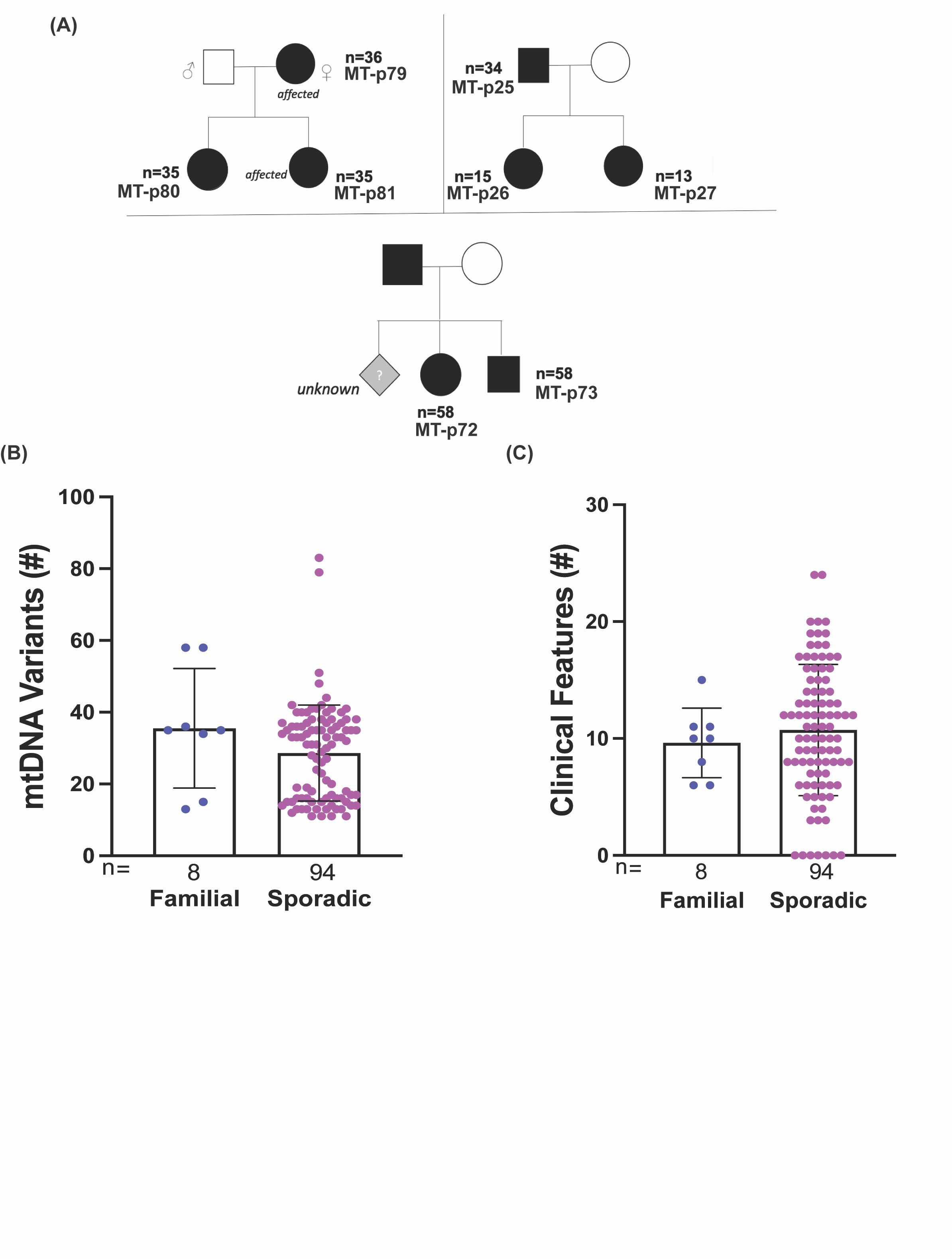

Supplement: Supplementary file 3 [file Image2.jpeg]
